# The genome sequence of the Brindled Ochre moth, *Dasypolia templi *(Thunberg, 1792)

**DOI:** 10.12688/wellcomeopenres.23054.1

**Published:** 2024-09-20

**Authors:** Andy Griffiths, Wellcome Sanger Institute Tree of Life Management, Samples and Laboratory team, Wellcome Sanger Institute Scientific Operations: Sequencing Operations, Wellcome Sanger Institute Tree of Life Core Informatics team, Tree of Life Core Informatics collective, Darwin Tree of Life Consortium

**Affiliations:** 1 Wellcome Sanger Institute, Hinxton, England, UK; 2 Royal Botanic Garden Edinburgh, Edinburgh, Scotland, UK

**Keywords:** Dasypolia templi, Brindled Ochre moth, genome sequence, chromosomal, Lepidoptera

## Abstract

We present a genome assembly from an individual male Brindled Ochre moth, *Dasypolia templi* (Arthropoda; Insecta; Lepidoptera; Noctuidae). The genome sequence has a total length of 855.30 megabases. Most of the assembly is scaffolded into 31 chromosomal pseudomolecules, including the Z sex chromosome. The mitochondrial genome has also been assembled and is 15.37 kilobases in length.

## Species taxonomy

Eukaryota; Opisthokonta; Metazoa; Eumetazoa; Bilateria; Protostomia; Ecdysozoa; Panarthropoda; Arthropoda; Mandibulata; Pancrustacea; Hexapoda; Insecta; Dicondylia; Pterygota; Neoptera; Endopterygota; Amphiesmenoptera; Lepidoptera; Glossata; Neolepidoptera; Heteroneura; Ditrysia; Obtectomera; Noctuoidea; Noctuidae; Cuculliinae; *Dasypoli*a; *Dasypolia templi* (Thunberg, 1792) (NCBI:txid987919).

## Background

**Figure 1.**
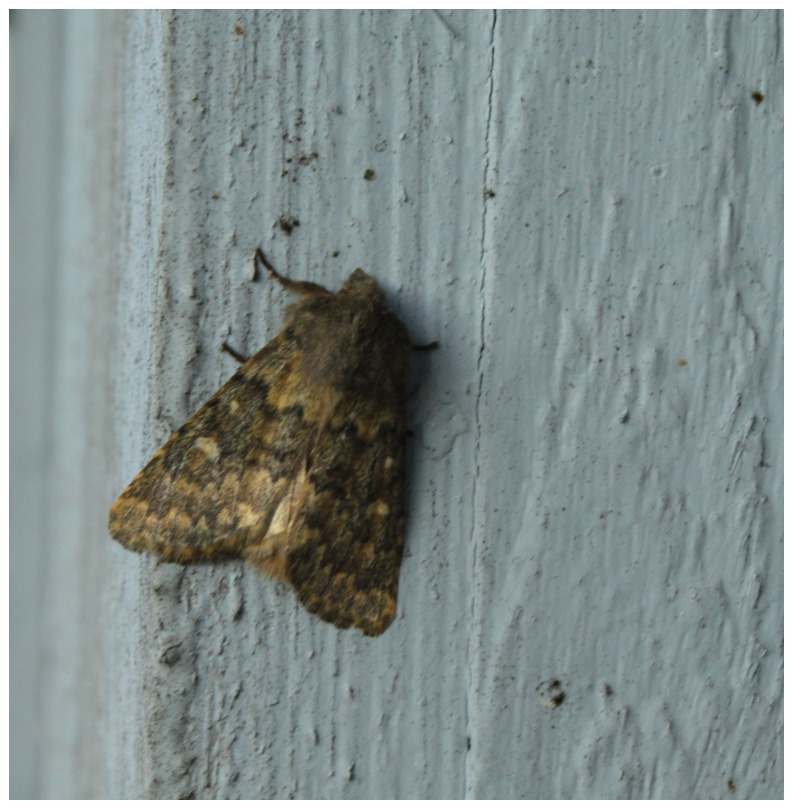
Photograph of *Dasypolia templi* by QWerk (not the specimen used for genome sequencing).

The Brindled Ochre moth, *Dasypolia templi* ( Figure 1), has a distribution that spans across Europe and parts of Asia. This species has been recorded in various countries, including the United Kingdom, France, Germany, and across Scandinavia, extending into Russia ( GBIF Secretariat, 2024). In the UK and Ireland, *D. templi* is found along the south-west coasts of England and Wales, and sporadically inland as well as coastally from the Midlands northwards through Scotland ( NBN Atlas Partnership, 2024).

The adults fly in one generation from September to early November in the UK and Ireland ( Waring *et al.*, 2017). Mating occurs in autumn, and the female overwinters, laying eggs on the larval foodplant, hogweed ( *Heracleum sphondylium*) and wild angelica ( *Angelica sylvestris*). Larvae appear in April to August, burrowing into the roots of the plant and pupating below the soil surface ( Waring *et al.*, 2017).

Here we present a chromosomally complete genome sequence for *Dasypolia templi*, based on a male specimen collected from the Southern Uplands in Scotland as part of the Darwin Tree of Life project.

## Genome sequence report

The genome of an adult *Dasypolia templi*was sequenced using Pacific Biosciences single-molecule HiFi long reads, generating a total of 81.88 Gb (gigabases) from 8.26 million reads, providing approximately 94-fold coverage. Primary assembly contigs were scaffolded with chromosome conformation Hi-C data, which produced 137.18 Gbp from 908.45 million reads, yielding an approximate coverage of 160-fold. Specimen and sequencing information is summarised in Table 1.

**Table 1.** Specimen and sequencing data for *Dasypolia templi*.

Project information			
**Study title**	Dasypolia templi (brindled ochre)		
**Umbrella BioProject**	PRJEB65412		
**Species**	*Dasypolia templi*		
**BioSample**	SAMEA112819851		
**NCBI taxonomy ID**	987919		
Specimen information			
**Technology**	**ToLID**	**BioSample accession**	**Organism part**
**PacBio long read sequencing**	ilDasTemp1	SAMEA112819909	thorax
**Hi-C sequencing**	ilDasTemp2	SAMEA112819900	head
Sequencing information			
**Platform**	**Run accession**	**Read count**	**Base count (Gb)**
**Hi-C Illumina NovaSeq 6000**	ERR11904132	9.08e+08	137.18
**PacBio Revio**	ERR11892490	8.26e+06	81.88

**Figure 2.**
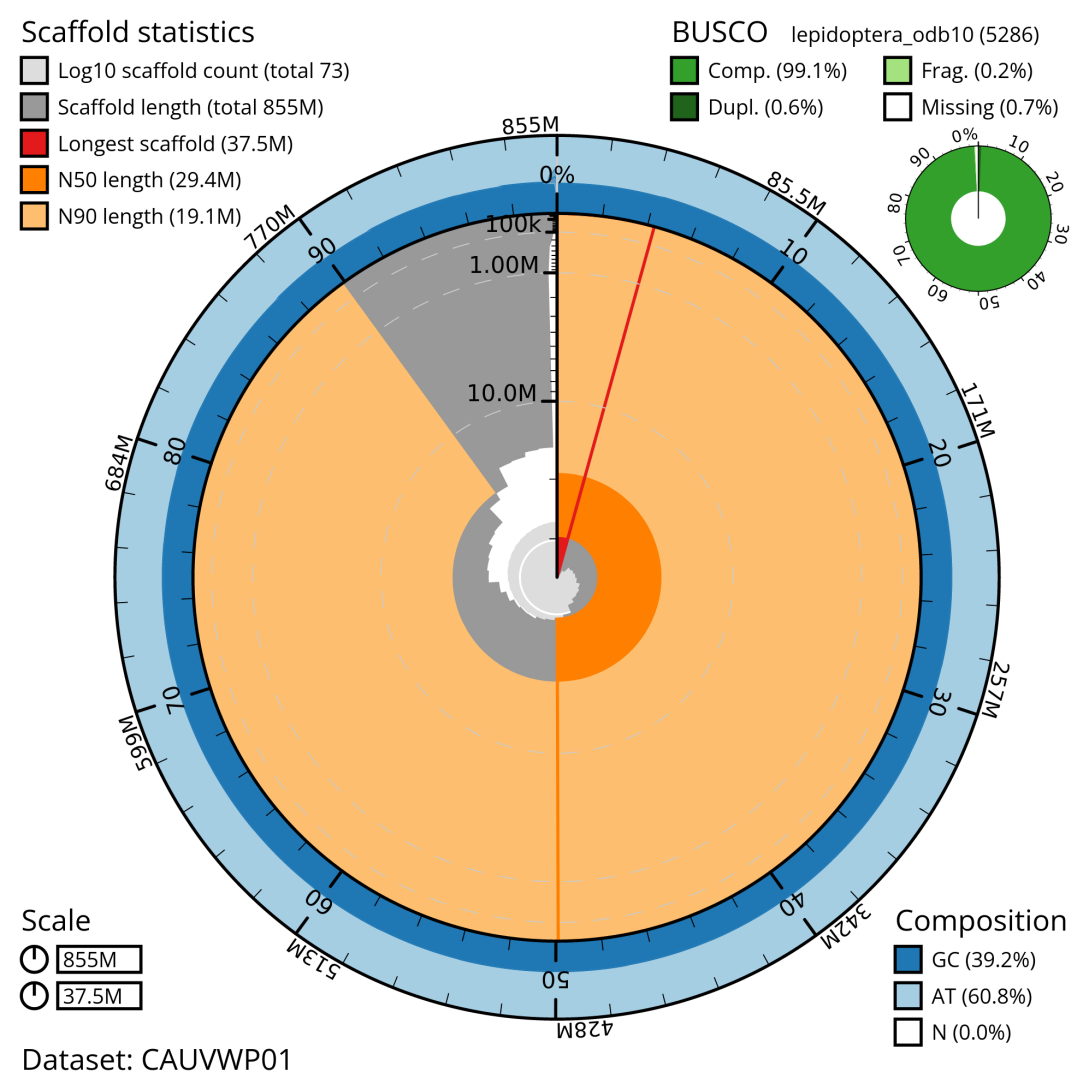
Genome assembly of *Dasypolia templi*, ilDasTemp1.1: metrics. The BlobToolKit snail plot shows N50 metrics and BUSCO gene completeness. The main plot is divided into 1,000 size-ordered bins around the circumference with each bin representing 0.1% of the 855,285,605 bp assembly. The distribution of scaffold lengths is shown in dark grey with the plot radius scaled to the longest scaffold present in the assembly (37,498,354 bp, shown in red). Orange and pale-orange arcs show the N50 and N90 scaffold lengths (29,373,807 and 19,078,010 bp), respectively. The pale grey spiral shows the cumulative scaffold count on a log scale with white scale lines showing successive orders of magnitude. The blue and pale-blue area around the outside of the plot shows the distribution of GC, AT and N percentages in the same bins as the inner plot. A summary of complete, fragmented, duplicated and missing BUSCO genes in the lepidoptera_odb10 set is shown in the top right. An interactive version of this figure is available at https://blobtoolkit.genomehubs.org/view/CAUVWP01/dataset/CAUVWP01/snail.

**Figure 3.**
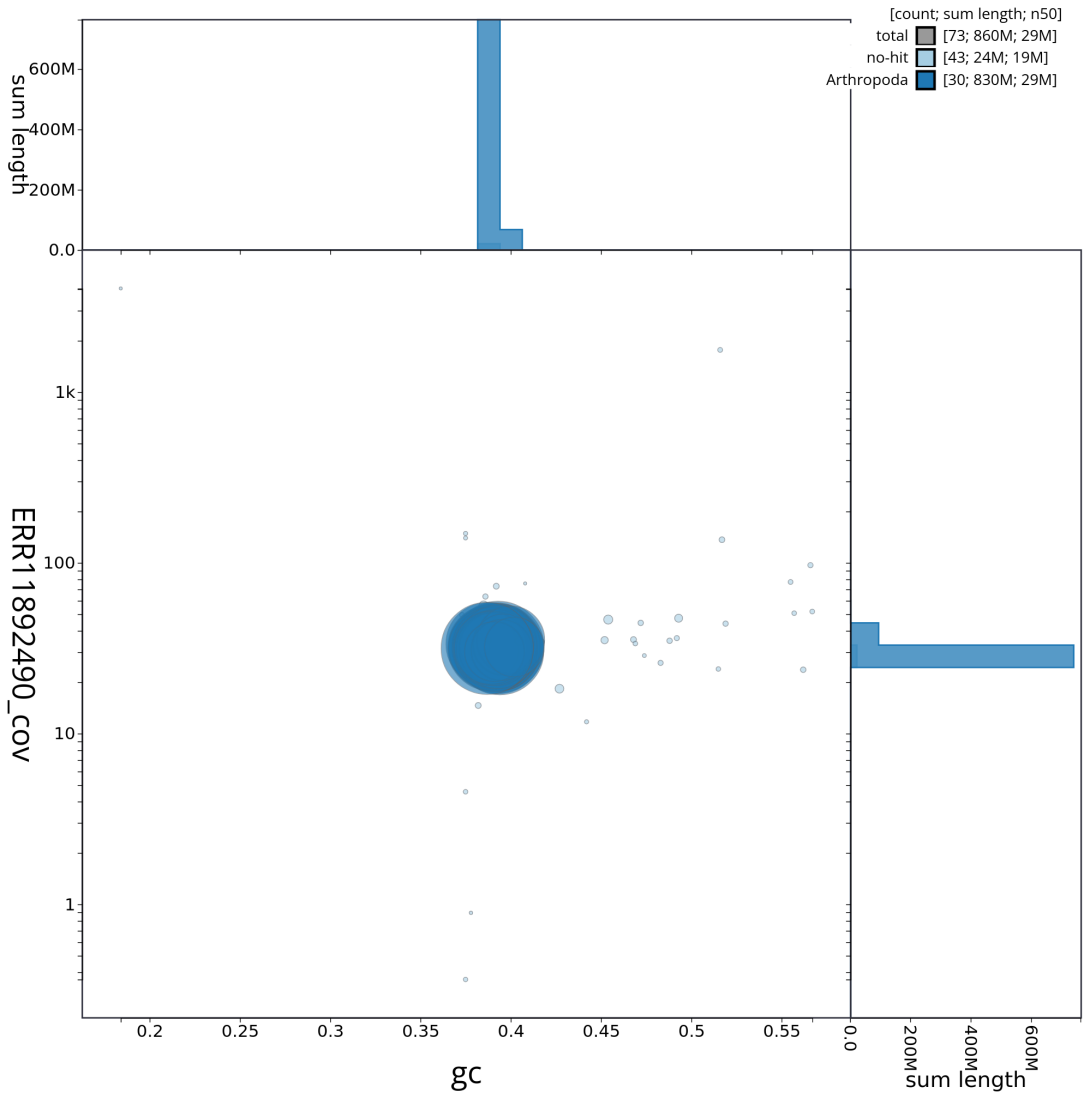
Genome assembly of *Dasypolia templi*, ilDasTemp1.1: BlobToolKit GC-coverage plot. Base coverage on the vertical axis is plotted against GC content on the horizontal axis. Sequences are coloured by phylum. Circles are sized in proportion to sequence length. Histograms show the distribution of sequence length sum along each axis. An interactive version of this figure is available at https://blobtoolkit.genomehubs.org/view/CAUVWP01/dataset/CAUVWP01/blob.

**Figure 4.**
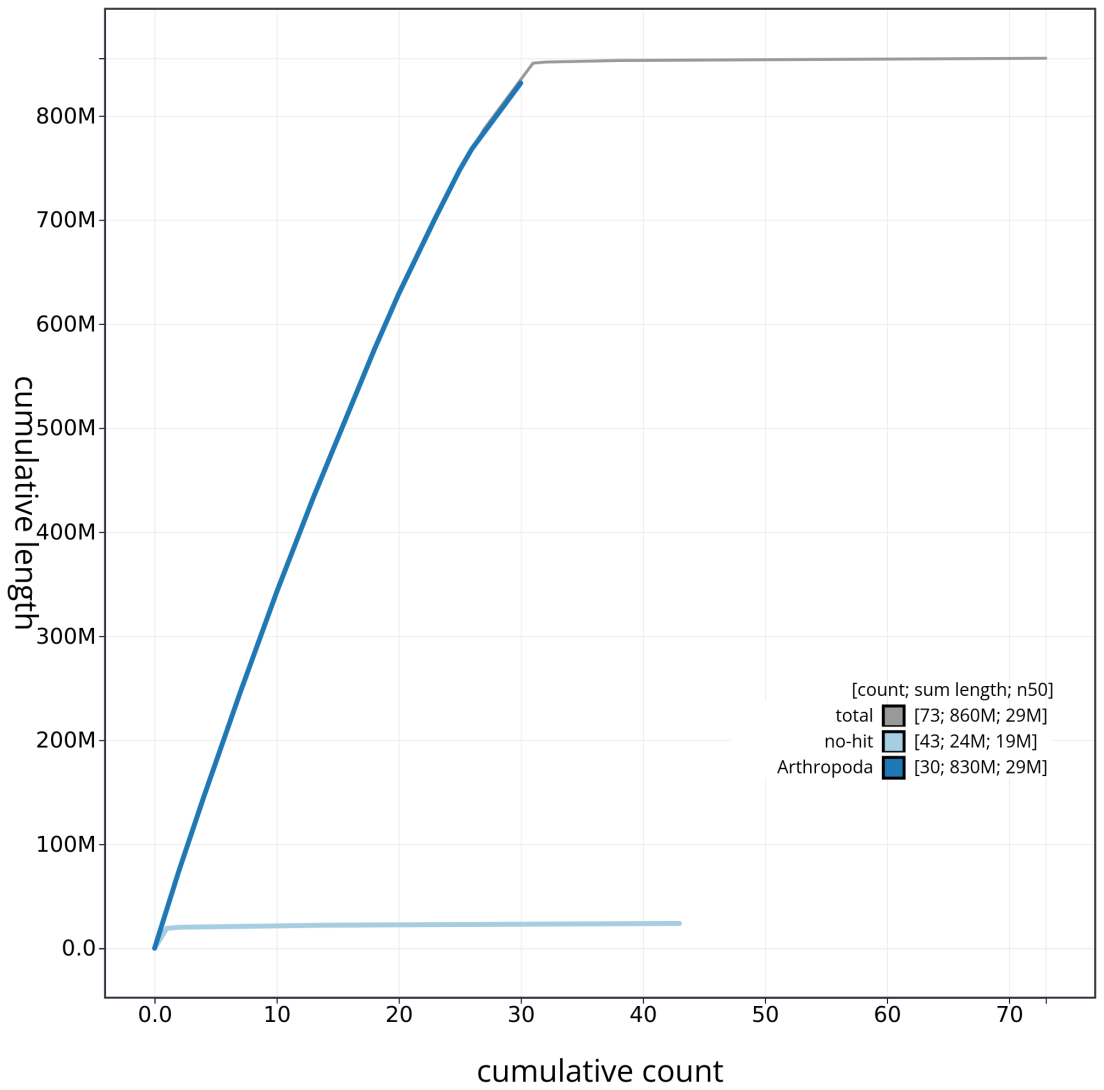
Genome assembly of *Dasypolia templi* ilDasTemp1.1: BlobToolKit cumulative sequence plot. The grey line shows cumulative length for all sequences. Coloured lines show cumulative lengths of sequences assigned to each phylum using the buscogenes taxrule. An interactive version of this figure is available at https://blobtoolkit.genomehubs.org/view/CAUVWP01/dataset/CAUVWP01/cumulative.

**Figure 5.**
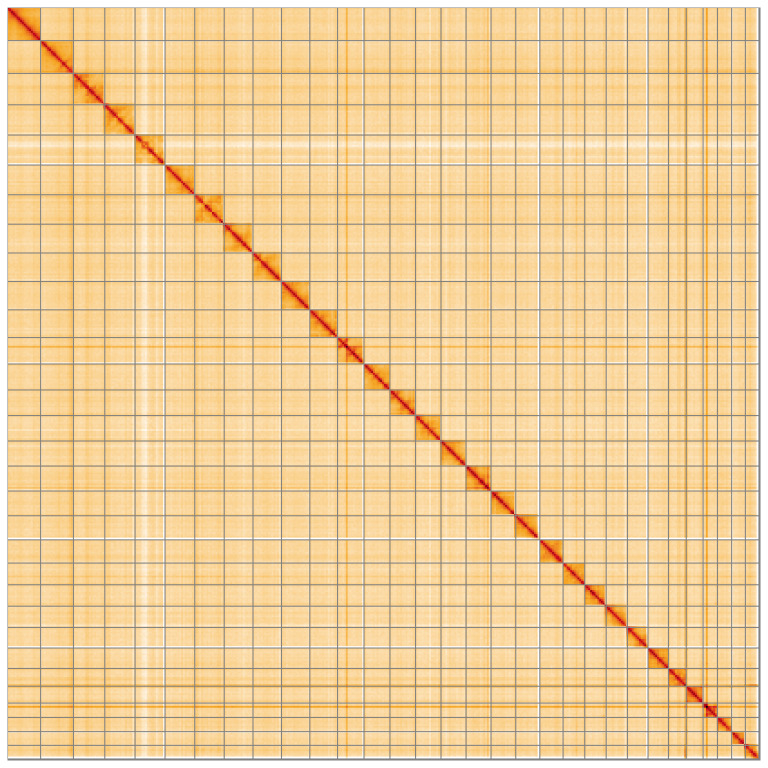
Genome assembly of *Dasypolia templi* ilDasTemp1.1: Hi-C contact map of the ilDasTemp1.1 assembly, visualised using HiGlass. Chromosomes are shown in order of size from left to right and top to bottom. An interactive version of this figure may be viewed at https://genome-note-higlass.tol.sanger.ac.uk/l/?d=C25BQ8UbSNCROgpV8FuuLg.

Manual assembly curation corrected 29 missing joins or mis-joins and 7 haplotypic duplications, reducing the assembly length by 0.35% and the scaffold number by 1.35%, and increasing the scaffold N50 by 0.48%. The final assembly has a total length of 855.30 Mb in 72 sequence scaffolds with a scaffold N50 of 29.4 Mb ( Table 2). The total count of gaps in the scaffolds is 70. The snail plot in Figure 2 provides a summary of the assembly statistics, while the distribution of assembly scaffolds on GC proportion and coverage is shown in Figure 3. The cumulative assembly plot in Figure 4 shows curves for subsets of scaffolds assigned to different phyla. Most (99.44%) of the assembly sequence was assigned to 31 chromosomal-level scaffolds, representing 30 autosomes and the Z sex chromosome. Chromosome-scale scaffolds confirmed by the Hi-C data are named in order of size ( Figure 5; Table 3). Chromosome Z was assigned by synteny to *Lithophane leautieri* (GCA_949152455.1) ( Lees *et al.*, 2024). While not fully phased, the assembly deposited is of one haplotype. Contigs corresponding to the second haplotype have also been deposited. The mitochondrial genome was also assembled and can be found as a contig within the multifasta file of the genome submission.

**Table 2.** Genome assembly data for *Dasypolia templi*, ilDasTemp1.1.

Genome assembly		
Assembly name	ilDasTemp1.1	
Assembly accession	GCA_963555695.1	
*Accession of alternate haplotype*	*GCA_963555655.1*	
Span (Mb)	855.30	
Number of contigs	143	
Contig N50 length (Mb)	13.2	
Number of scaffolds	72	
Scaffold N50 length (Mb)	29.4	
Longest scaffold (Mb)	37.5	
Assembly metrics *		*Benchmark*
Consensus quality (QV)	67.4	*≥ 50*
*k*-mer completeness	100.0%	*≥ 95%*
BUSCO **	C:99.1%[S:98.5%,D:0.6%],F:0.2%,M:0.7%,n:5,286	*C ≥ 95%*
Percentage of assembly mapped to chromosomes	99.44%	*≥ 95%*
Sex chromosomes	Z	*localised homologous pairs*
Organelles	Mitochondrial genome: 15.37 kb	*complete single alleles*

* Assembly metric benchmarks are adapted from column VGP-2020 of “Table 1: Proposed standards and metrics for defining genome assembly quality” from Rhie *et al.* (2021). ** BUSCO scores based on the lepidoptera_odb10 BUSCO set using version 5.3.2. C = complete [S = single copy, D = duplicated], F = fragmented, M = missing, n = number of orthologues in comparison. A full set of BUSCO scores is available at https://blobtoolkit.genomehubs.org/view/CAUVWP01/dataset/CAUVWP01/busco.

**Table 3.** Chromosomal pseudomolecules in the genome assembly of *Dasypolia templi*, ilDasTemp1.

INSDC accession	Name	Length (Mb)	GC%
OY743137.1	1	37.12	39.5
OY743138.1	2	35.52	39.5
OY743139.1	3	34.24	39.0
OY743140.1	4	33.93	39.5
OY743141.1	5	33.65	39.0
OY743142.1	6	33.34	39.0
OY743143.1	7	32.58	39.0
OY743144.1	8	32.25	39.5
OY743145.1	9	32.07	39.0
OY743146.1	10	31.42	39.0
OY743147.1	11	29.91	38.5
OY743148.1	12	29.37	39.0
OY743149.1	13	29.01	39.0
OY743150.1	14	28.79	39.0
OY743151.1	15	28.34	39.0
OY743152.1	16	28.28	39.0
OY743153.1	17	28.0	39.0
OY743154.1	18	27.22	39.5
OY743155.1	19	26.31	39.5
OY743156.1	20	24.56	39.0
OY743157.1	21	24.25	39.0
OY743158.1	22	24.04	39.0
OY743159.1	23	23.3	39.0
OY743160.1	24	23.1	39.0
OY743161.1	25	20.16	40.0
OY743162.1	26	19.08	38.5
OY743163.1	27	16.23	39.5
OY743164.1	28	16.11	39.5
OY743165.1	29	15.51	39.0
OY743166.1	30	15.38	40.0
OY743136.1	Z	37.5	38.5
OY743167.1	MT	0.02	18.5

The estimated Quality Value (QV) of the final assembly is 67.4 with *k*-mer completeness of 100.0%, and the assembly has a BUSCO v5.3.2 completeness of 99.1% (single = 98.5%, duplicated = 0.6%), using the lepidoptera_odb10 reference set ( *n* = 5,286).

Metadata for specimens, BOLD barcode results, spectra estimates, sequencing runs, contaminants and pre-curation assembly statistics are given at https://links.tol.sanger.ac.uk/species/987919.

## Methods

### Sample acquisition

Adult specimens of *Dasypolia templi* were collected from Gameshope, Moffat Hills, Southern Uplands, Scotland, UK (latitude 55.45, longitude –3.37) on 2022-09-24. The specimens were collected and identified by Andy Griffiths (Wellcome Sanger Institute). Specimen ID SAN00002760 (ToLID ilDasTemp1) was used for PacBio HiFi sequencing and specimen ID SAN00002756 (ToLID ilDasTemp2) was used for Hi-C sequencing.

The initial identification was verified by an additional DNA barcoding process according to the framework developed by Twyford *et al*. (2024). A small sample was dissected from the specimens and stored in ethanol, while the remaining parts of the specimen were shipped on dry ice to the Wellcome Sanger Institute (WSI). The tissue was lysed, the COI marker region was amplified by PCR, and amplicons were sequenced and compared to the BOLD database, confirming the species identification ( Crowley *et al.*, 2023). Following whole genome sequence generation, the relevant DNA barcode region is also used alongside the initial barcoding data for sample tracking at the WSI ( Twyford *et al.*, 2024). The standard operating procedures for Darwin Tree of Life barcoding have been deposited on protocols.io ( Beasley *et al.*, 2023).

### Nucleic acid extraction

The workflow for high molecular weight (HMW) DNA extraction at the Wellcome Sanger Institute (WSI) Tree of Life Core Laboratory includes a sequence of core procedures: sample preparation and homogenisation, DNA extraction, fragmentation and purification. Detailed protocols are available on protocols.io ( Denton *et al.*, 2023b). The ilDasTemp1 sample was weighed and dissected on dry ice ( Jay *et al.*, 2023). Tissue from the thorax was homogenised using a PowerMasher II tissue disruptor ( Denton *et al.*, 2023a).

HMW DNA was extracted in the WSI Scientific Operations core using the Automated MagAttract v2 protocol ( Oatley *et al.*, 2023). The DNA was sheared into an average fragment size of 12–20 kb in a Megaruptor 3 system ( Bates *et al.*, 2023). Sheared DNA was purified by solid-phase reversible immobilisation, using AMPure PB beads to eliminate shorter fragments and concentrate the DNA ( Strickland *et al.*, 2023). The concentration of the sheared and purified DNA was assessed using a Nanodrop spectrophotometer and Qubit Fluorometer using the Qubit dsDNA High Sensitivity Assay kit. Fragment size distribution was evaluated by running the sample on the FemtoPulse system.

### Library preparation and sequencing

Pacific Biosciences HiFi circular consensus DNA sequencing libraries were constructed according to the manufacturers’ instructions. DNA sequencing was performed by the Scientific Operations core at the WSI on a Pacific Biosciences Revio instrument.

Hi-C data were generated from frozen head tissue of the ilDasTemp2 sample, using the Arima-HiC v2 kit. The tissue was fixed with a TC buffer containing formaldehyde, resulting in crosslinked DNA. The crosslinked DNA was digested with a restriction enzyme master mix. The resulting 5’-overhangs were filled in and labelled with a biotinylated nucleotide. The biotinylated DNA was then fragmented, enriched, barcoded, and amplified using the NEBNext Ultra II DNA Library Prep Kit. Hi-C sequencing was performed on an Illumina NovaSeq 6000 instrument, using paired-end sequencing with a read length of 150 bp.

### Genome assembly, curation and evaluation

***Assembly***

The HiFi reads were first assembled using Hifiasm ( Cheng *et al.*, 2021) with the --primary option. Haplotypic duplications were identified and removed using purge_dups ( Guan *et al.*, 2020). The Hi-C reads were mapped to the primary contigs using bwa-mem2 ( Vasimuddin *et al.*, 2019). The contigs were further scaffolded using the provided Hi-C data ( Rao *et al.*, 2014) in YaHS ( Zhou *et al.*, 2023) using the --break option. The scaffolded assemblies were evaluated using Gfastats ( Formenti *et al.*, 2022), BUSCO ( Manni *et al.*, 2021) and MERQURY.FK ( Rhie *et al.*, 2020).

The mitochondrial genome was assembled using MitoHiFi ( Uliano-Silva *et al.*, 2023), which runs MitoFinder ( Allio *et al.*, 2020) and uses these annotations to select the final mitochondrial contig and to ensure the general quality of the sequence.

***Assembly curation***

The assembly was decontaminated using the Assembly Screen for Cobionts and Contaminants (ASCC) pipeline (article in preparation). Flat files and maps used in curation were generated in TreeVal ( Pointon *et al.*, 2023). Manual curation was primarily conducted using PretextView ( Harry, 2022), with additional insights provided by JBrowse2 ( Diesh *et al.*, 2023) and HiGlass ( Kerpedjiev *et al.*, 2018). Scaffolds were visually inspected and corrected as described by Howe *et al*. (2021). Any identified contamination, missed joins, and mis-joins were corrected, and duplicate sequences were tagged and removed. The sex chromosome was identified by synteny analysis. The curation process is documented at https://gitlab.com/wtsi-grit/rapid-curation (article in preparation).

***Evaluation of the final assembly***

A Hi-C map for the final assembly was produced using bwa-mem2 ( Vasimuddin *et al.*, 2019) in the Cooler file format ( Abdennur & Mirny, 2020). To assess the assembly metrics, the *k*-mer completeness and QV consensus quality values were calculated in Merqury ( Rhie *et al.*, 2020). This work was done using the “sanger-tol/readmapping” ( Surana *et al.*, 2023a) and “sanger-tol/genomenote” ( Surana *et al.*, 2023b) pipelines. The genome readmapping pipelines were developed using the nf-core tooling ( Ewels *et al.*, 2020), use MultiQC ( Ewels *et al.*, 2016), and make extensive use of the Conda package manager, the Bioconda initiative ( Grüning *et al.*, 2018), the Biocontainers infrastructure ( da Veiga Leprevost *et al.*, 2017), and the Docker ( Merkel, 2014) and Singularity ( Kurtzer *et al.*, 2017) containerisation solutions. The genome was also analysed within the BlobToolKit environment ( Challis *et al.*, 2020) and BUSCO scores ( Manni *et al.*, 2021; Simão *et al.*, 2015) were calculated.

Table 4 contains a list of relevant software tool versions and sources.

**Table 4.** Software tools: versions and sources.

Software tool	Version	Source
BlobToolKit	4.2.1	https://github.com/blobtoolkit/blobtoolkit
BUSCO	5.3.2	https://gitlab.com/ezlab/busco
bwa-mem2	2.2.1	https://github.com/bwa-mem2/bwa-mem2
Cooler	0.8.11	https://github.com/open2c/cooler
Gfastats	1.3.6	https://github.com/vgl-hub/gfastats
Hifiasm	0.19.5-r587	https://github.com/chhylp123/hifiasm
HiGlass	1.11.6	https://github.com/higlass/higlass
Merqury.FK	d00d98157618f4e8d1a9190026b19b471055b22e	https://github.com/thegenemyers/MERQURY.FK
MitoHiFi	3	https://github.com/marcelauliano/MitoHiFi
PretextView	0.2	https://github.com/wtsi-hpag/PretextView
purge_dups	1.2.5	https://github.com/dfguan/purge_dups
sanger-tol/genomenote	v1.0	https://github.com/sanger-tol/genomenote
sanger-tol/readmapping	1.1.0	https://github.com/sanger-tol/readmapping/tree/1.1.0
Singularity	3.9.0	https://github.com/sylabs/singularity
YaHS	1.2a.2	https://github.com/c-zhou/yahs

### Wellcome Sanger Institute – Legal and Governance

The materials that have contributed to this genome note have been supplied by a Darwin Tree of Life Partner. The submission of materials by a Darwin Tree of Life Partner is subject to the **‘Darwin Tree of Life Project Sampling Code of Practice’**, which can be found in full on the Darwin Tree of Life website here. By agreeing with and signing up to the Sampling Code of Practice, the Darwin Tree of Life Partner agrees they will meet the legal and ethical requirements and standards set out within this document in respect of all samples acquired for, and supplied to, the Darwin Tree of Life Project.

Further, the Wellcome Sanger Institute employs a process whereby due diligence is carried out proportionate to the nature of the materials themselves, and the circumstances under which they have been/are to be collected and provided for use. The purpose of this is to address and mitigate any potential legal and/or ethical implications of receipt and use of the materials as part of the research project, and to ensure that in doing so we align with best practice wherever possible. The overarching areas of consideration are:

•   Ethical review of provenance and sourcing of the material

•   Legality of collection, transfer and use (national and international) 

Each transfer of samples is further undertaken according to a Research Collaboration Agreement or Material Transfer Agreement entered into by the Darwin Tree of Life Partner, Genome Research Limited (operating as the Wellcome Sanger Institute), and in some circumstances other Darwin Tree of Life collaborators.

## Data Availability

European Nucleotide Archive: Dasypolia templi (brindled ochre). Accession number PRJEB65412; https://identifiers.org/ena.embl/PRJEB65412 ( Wellcome Sanger Institute, 2023). The genome sequence is released openly for reuse. The *Dasypolia templi* genome sequencing initiative is part of the Darwin Tree of Life (DToL) project. All raw sequence data and the assembly have been deposited in INSDC databases. The genome will be annotated using available RNA-Seq data and presented through the Ensembl pipeline at the European Bioinformatics Institute. Raw data and assembly accession identifiers are reported in Table 1 and Table 2.

## Author information

Members of the Wellcome Sanger Institute Tree of Life Management, Samples and Laboratory team are listed here: https://doi.org/10.5281/zenodo.12162482.

Members of Wellcome Sanger Institute Scientific Operations: Sequencing Operations are listed here: https://doi.org/10.5281/zenodo.12165051.

Members of the Wellcome Sanger Institute Tree of Life Core Informatics team are listed here: https://doi.org/10.5281/zenodo.12160324.

Members of the Tree of Life Core Informatics collective are listed here: https://doi.org/10.5281/zenodo.12205391.

Members of the Darwin Tree of Life Consortium are listed here: https://doi.org/10.5281/zenodo.4783558.
