# Supplementary material for: The genome sequence of the Brindled Ochre moth, Dasypolia templi (Thunberg, 1792)
Source: Wellcome Open Res. 2024 Sep 20;9:542. [Version 1] doi: 10.12688/wellcomeopenres.23054.1 (PMC11525101; doi:10.12688/wellcomeopenres.23054.1)
Supplement: Peer review history

# Reviewer report: Reviewer response for version 1

**DOI:** 10.21956/wellcomeopenres.25390.r102428

**Published:** 2024-10-30

**Authors:** Jisheng Liu

**Affiliations:** 1 Guangzhou University, Guangzhou, China

This manuscript reports the genome sequence of an individual male Brindled Ochre moth, *Dasypolia templi*, which includes the Z sex chromosome and the mitochondrial genome. The methods are clearly described and the data are clearly presented in the manuscript.

 However, there are some questions that need to be answered and discussed in the manuscript.

 1. What is the importance to sequence the genome of this moth?

 2. Why a male moth was chosen instead of a female one? The female moth includes the Z and W sex chromosomes, which might provide more genome information.

 3. Since the silkmoth *Bombyx mori* is the model insect in Lepidoptera. Could the author compare the genomes among the *Dasypolia templi* and *Bombyx mori* to show the shared and unique information?

Are sufficient details of methods and materials provided to allow replication by others?

Yes

Is the rationale for creating the dataset(s) clearly described?

Partly

Are the datasets clearly presented in a useable and accessible format?

Yes

Are the protocols appropriate and is the work technically sound?

Yes

Reviewer Expertise:

Insect biochemistry, insect molecular biology, insect physiology

I confirm that I have read this submission and believe that I have an appropriate level of expertise to confirm that it is of an acceptable scientific standard, however I have significant reservations, as outlined above.

# Reviewer report: Reviewer response for version 1

**DOI:** 10.21956/wellcomeopenres.25390.r103550

**Published:** 2024-10-28

**Authors:** Yu-Feng Huang

**Affiliations:** 1 National Chung Hsing University, Taichung, Taiwan

The manuscript titled “The Genome Sequence of the Brindled Ochre Moth, *Dasypolia templi* (Thunberg, 1792)” presents a high-quality genome assembly of *Dasypolia templi*, developed as part of the Darwin Tree of Life Project. Utilizing Pacific Biosciences HiFi long reads alongside Illumina Hi-C short reads, the authors achieved an assembly of 855.30 megabases, organized into 31 chromosomal pseudomolecules, which notably includes the Z sex chromosome. This work significantly advances our understanding of the genomic structure of this moth species and includes the complete mitochondrial genome.

 The technical merits of the study are commendable, as the genome assembly demonstrates both high accuracy and completeness. With a scaffold N50 of 29.4 megabases and a BUSCO completeness score of 99.1%, the assembly is of exceptional quality. The methodology is well-articulated, detailing the sequencing processes, bioinformatics tools employed, and quality control measures. The use of tools such as Merqury for k-mer completeness assessment and BUSCO for evaluating genome completeness adds to the rigor of the study. Furthermore, the open-access nature of the data enhances the study’s value, facilitating reuse and exploration by other researchers.

 While the paper provides an impressive genome assembly, the impact of the work could be further strengthened by incorporating additional biological analyses. It serves as a solid foundation for future research into the genetics and ecology of *Dasypolia templi* and related species.

**However, several key areas require clarification to enhance the clarity and overall impact of the manuscript:**

- First, the authors should explicitly confirm whether the circularity of the mitochondrial genome has been validated. This clarification is essential for readers to fully understand the assembly process.
- Second, there is inconsistency in the terminology used when referring to tools for assembly metrics. Both "MERQURY.FY" and "Merqury" appear in the text, which could lead to confusion. Consistent terminology should be maintained throughout the manuscript.
- Moreover, the authors should not only list the tools utilized but also provide insight into the rationale behind their selection and how they were integrated into the assembly and evaluation process.
- Additionally, the "Evaluation of the Final Assembly" section would benefit from a more organized presentation. For example, while nf-core is identified as an open-source platform for pipelines developed with the Next flow framework, Docker and Singularity serve as containerization solutions, and Conda and Bioconda provide virtual environments. Rather than merely enumerating these tools, the authors should elaborate on how they were cohesively employed in a practical workflow. This would enhance the accessibility of the methodology and offer valuable insights into the feasibility and replicability of the approaches taken in this project.

Are sufficient details of methods and materials provided to allow replication by others?

Yes

Is the rationale for creating the dataset(s) clearly described?

Yes

Are the datasets clearly presented in a useable and accessible format?

Yes

Are the protocols appropriate and is the work technically sound?

Yes

Reviewer Expertise:

genome assembly, annotation and evaluation; bioinformatics; cancer genome biology;

I confirm that I have read this submission and believe that I have an appropriate level of expertise to confirm that it is of an acceptable scientific standard, however I have significant reservations, as outlined above.

# Reviewer report: Reviewer response for version 1

**DOI:** 10.21956/wellcomeopenres.25390.r104629

**Published:** 2024-10-18

**Authors:** Thomas Brown

**Affiliations:** 1 Leibniz IZW (Berlin) & ERGA/BGP, Leibniz, Germany

Griffiths and the Darwin Tree of Life team present a complete genome assembly of the moth *Dasypolia templi*. In general, the assembly and the methods used to generate the reference are of high quality and well documented.

**I highlight below a few areas that should be addressed:**

**Background:**

 It is not stated why having a reference genome is of importance or note for this species. What is this resource contributing to the field that was previously lacking?

**Figure 1:**

 Please add a link to the licence, as is stated in the re-use statement from Wikipedia: https://creativecommons.org/licenses/by-sa/3.0/deed.en

**Table 1 and sampling information:**

 It appears from the BioSamples and ToLIDs that this assembly was generated from two individuals, but this is not overly clear from the text. It is also a little confusing to have a separate BioSample for the assembly from which the samples used for PacBio and Illumina were derived. The reader would benefit from more clarification in this area.

**Methods:**

 The "Assembly Screen for Cobionts and Contaminants (ASCC) pipeline" is stated as having an article in preparation, but a link to the pipeline and relevant code should be included in this manuscript.

 Otherwise I am happy that the metadata and data are clearly presented and that all data and outputs are of high quality.

Are sufficient details of methods and materials provided to allow replication by others?

Yes

Is the rationale for creating the dataset(s) clearly described?

Partly

Are the datasets clearly presented in a useable and accessible format?

Yes

Are the protocols appropriate and is the work technically sound?

Yes

Reviewer Expertise:

Genome Assembly, Genome Annotation

I confirm that I have read this submission and believe that I have an appropriate level of expertise to confirm that it is of an acceptable scientific standard.
